# Examining job involvement and perceived organizational support toward organizational commitment: job insecurity as mediator

**DOI:** 10.3389/fpsyg.2024.1290122

**Published:** 2024-01-29

**Authors:** Chin Ling Hngoi, Nurul-Azza Abdullah, Wan Shahrazad Wan Sulaiman, Norshaffika Izzaty Zaiedy Nor

**Affiliations:** Faculty of Social Science and Humanities, Universiti Kebangsaan Malaysia, Bangi, Malaysia

**Keywords:** job involvement, perceived organizational support, job insecurity, organizational commitment, employment flexibility, positions (in)security, tenure (in)security

## Abstract

This study delves into the intricate relationships among job involvement (JI), perceived organizational support (POS), job insecurity (JIS), and organizational commitment (OC), with a particular focus on the mediating role of JIS within the context of the Malaysian private sector. The research delves into the antecedents of job insecurity and organizational commitment, offering insights to enhance commitment. Our study involved 440 employees in the Malaysian private sector, utilizing self-report questionnaires administered online. Notably, our findings underscore the significance of employment flexibility, job positions, and tenure in shaping JIS. Furthermore, we identify significant relationships among the variables: POS negatively predicts JIS, while JI, JIS, and POS collectively predict OC, with JIS partially mediating the POS-OC relationship. These empirically-grounded insights offer actionable guidance for organizations, empowering human resources practitioners to craft effective talent retention strategies and allocate resources strategically. In doing so, organizations can enhance employee productivity and bolster organizational commitment, ultimately contributing to sustained success in a dynamic work environment. These findings hold valuable implications for human resources practitioners, guiding the development of talent retention strategies and resource allocation to enhance employee productivity.

## 1 Introduction

The Fourth Industrial Revolution has significantly transformed how organizations and individuals operate, driven by digitalization and technological advancements. These changes have increased competitiveness and presented new challenges, further exacerbated by the COVID-19 pandemic. In response to these dynamic circumstances, organizations have adopted innovative strategies such as downsizing, restructuring, and flexible employment to ensure sustainability. The availability of low-cost airlines in developing regions like Southeast Asia has facilitated increased workforce mobility and cross-country management, leading to the emergence of common headquarters in cities like Singapore and Hong Kong for corporations managing localized business operations in the region (Hngoi et al., 2023).

These shifts in organizational strategies and structures have directly and indirectly influenced the content and structure of work, placing increased pressure and uncertainties on employees. Graduates, in particular, have been significantly affected, facing rising unemployment due to shifting skill demands and market niches (Omar et al., 2012; Sarfraz et al., 2018). Additionally, organizational changes contribute to job insecurity among employees, with job involvement and perceived organizational support playing crucial roles as predictors. Job insecurity, in turn, negatively impacts employee commitment and productivity (De Cuyper and De Witte, 2005; Kraimer et al., 2005; Chen et al., 2007; Green and Leeves, 2013; Wilczyńska et al., 2015; Shoss, 2017; Filimonau et al., 2020). Furthermore, the recent shift toward employment flexibility, remote work, and hybrid working models has significantly influenced the dynamics of job roles. With the advent of technology and the need for adaptability, organizations have increasingly embraced these flexible work arrangements. While they offer benefits such as improved work-life balance and increased autonomy, they also introduce new challenges for employees.

Understanding the factors influencing organizational success and how they impact workforce dynamics and productivity has become imperative for organizations. Researchers have explored various variables influencing organizational behavior, including the relationship between organizations and employees, which affects job performance, productivity, colleague relationships, and society (Mowday et al., 1982; Sverke et al., 2019; Ridwan et al., 2020; Shao et al., 2022). While early research predominantly focused on job satisfaction, attention gradually shifted toward studying other attitudinal concepts such as job involvement, perceived organizational support, and organizational commitment. Extensive literature reviews have highlighted the importance of studying the antecedents and predictors that shape organizational behavior, with over 70 studies examining workplace attitudes (Rhoades and Eisenberger, 2002; Shoss, 2017; Saeed et al., 2023). Leadership, interpersonal interactions, company culture, and relationship dynamics have been identified as influential factors.

Job insecurity, a concept first studied in the early 1990s, carries significant implications for employees and is considered a critical aspect of work quality (Roskies and Louis-Guerin, 1990; Manski and Straub, 2000). It can be defined differently, such as objective circumstances or perceptual phenomena (Greenhalgh and Rosenblatt, 1984; Bordia et al., 2004). Job insecurity negatively affects employees’ physical wellbeing, mental health, and job satisfaction (De Witte, 1999; Kurnia and Widigdo, 2021). It can be categorized into qualitative and quantitative forms (Hellgren et al., 1999; Hellgren and Sverke, 2003). The latent deprivation model suggests that job insecurity creates hidden deprivation beyond financial constraints, affecting individuals’ sense of identity and self-worth (Jahoda, 1981, 1982). The vitamin model emphasizes the importance of work environment characteristics for employee wellbeing (Warr, 1987, 1990). Job insecurity has been found to lead to decreased job satisfaction and performance, ultimately impacting organizational productivity (Mowday et al., 1979; Greenhalgh and Rosenblatt, 1984; Sverke et al., 2002). Consequently, job insecurity remains a hidden problem for organizations, as employees must navigate the uncertainties of job retention while fulfilling their work responsibilities. This burden negatively affects job satisfaction, individual performance, and organizational productivity (Greenhalgh and Rosenblatt, 1984; Jones et al., 1998; Filimonau et al., 2020).

To mitigate the adverse effects of job insecurity and foster organizational commitment, organizations should delve into the determinants of both phenomena and gain a deeper understanding of how perceived organizational support influences them. Additionally, studying management strategies to help employees cope with stress and job burnout resulting from organizational changes is essential (Shoss, 2017). These negative impacts on employees and organizational productivity have necessitated the exploration of work attitudes and demographic variables such as age, gender, educational background, and financial stability, which play a role in employee social changes within the organization and daily life (Mowday et al., 1982).

Organizational commitment, a crucial work attitude studied in organizational and industrial psychology, has been extensively researched over the past four decades. Numerous studies have established a significant relationship between organizational commitment and various organizational outcomes and employee productivity (Porter et al., 1974; Meyer and Allen, 1997; Chen et al., 2007; Ibrahim et al., 2018; Chow and Abdullah, 2019). Employee commitment is influenced by interaction experiences and perceptions of organizational support, job and organization stability, and employment security (Chen et al., 2007; Shoss, 2017; Salessi and Omar, 2019).

Despite the growing recognition of the importance of job insecurity, perceived organizational support, and job involvement in shaping organizational commitment; there exists a notable research gap in exploring the intricate dynamics among these variables. While studies have identified significant predictions, there is a dearth of research specifically addressing the mediating role of job insecurity in the relationships between the variables. Furthermore, the limited existing literature fails to comprehensively analyze job insecurity across demographic differences and organizational conditions, impeding the development of tailored policies and strategies for organizations.

This research aims to address these gaps by empirically examining the relationships between job involvement, job involvement, perceived organizational support, job insecurity, and organizational commitment, with a particular focus on the mediating effect of job insecurity. By investigating job insecurity based on demographic variations and organizational contexts, this study intends to contribute valuable insights to both academia and practitioners. Further to the systematic literature review conducted by Hngoi et al. (2023) this study seeks to provide valuable insights and practical guidance to addressing the research gap and contribution for organizations in effectively managing and promoting employee commitment. Understanding the factors that influence job insecurity and organizational commitment can assist organizations in developing targeted interventions and strategies to enhance employee resilience, job satisfaction, and organizational outcomes.

## 2 Job insecurity with positional characteristics

Research has been conducted on job insecurity with the positional characteristics of employment flexibility, position, and tenure within organizations, especially focusing on employment flexibility due to the COVID-19 outbreak (Adebayo, 2006; Blackmore and Kuntz, 2011; Ngo et al., 2013; Bohle et al., 2018; Mehta, 2022). Current research is skewed toward the mediation relationship between job insecurity and other variables of psychological wellbeing and other job attitudes, such as burnout, work engagement, wellbeing, emotional intelligence, and job satisfaction (Adebayo, 2006; Cheng and Chan, 2008; De Cuyper et al., 2008; Baillien and De Witte, 2009; Blackmore and Kuntz, 2011; Nasir et al., 2011; Safaria et al., 2011; Ouyang et al., 2014; Park and Ono, 2016; Bohle et al., 2018). Contrary to the prediction made by Shoss (2017) and Bose and Biswas (2019) found no significant relationship between position within the organization and job insecurity. The result implies that an employee’s hierarchical level or job title may not directly predict job insecurity. However, this finding highlights the need for further investigation into other positional characteristics that may contribute to job insecurity.

In addition to position within the organization, exploring the impact of employment flexibility on job insecurity is important, especially in the current post-pandemic. With the widespread adoption of remote work and the transition from traditional working arrangements to flexible practices such as hot-desking (Bose and Biswas, 2019), employees may experience heightened job insecurity. The uncertainty surrounding work location and the lack of a designated workspace can create instability and insecurity among employees.

Moreover, the duration of employment tenure within an organization is another positional characteristic that warrants investigation in relation to job insecurity. Long-tenured employees may perceive their job security differently than those relatively new to the organization. Understanding how tenure influences job insecurity can provide insights into the psychological contract between employees and employers, particularly regarding long-term job stability. Therefore, building on the literature review, we developed the first hypotheses in exploring the relationship between job insecurity and various positional characteristics, including employment flexibility, position within the organization, and tenure.

*H*1: Differences in job insecurity based on positional characteristics.

*H*1a: Job insecurity differs based on employment flexibility.

*H*1b: Job insecurity differs based on job positions.

*H*1c: Job insecurity differs based on tenure.

## 3 Job involvement, perceived organization support, and job insecurity

The relationship between perceived organizational support, job involvement, and job insecurity has been a subject of interest in organizational psychology (Clay-Warner et al., 2005; Singh and Gupta, 2014; Shoss, 2017; Mendoza, 2019; Salessi and Omar, 2019; Eisenberger et al., 2020). Job involvement refers to the degree to which employees are emotionally and cognitively engaged in their work, feeling a sense of meaning, purpose, and identification with their job (Blau and Meyer, 1987; Singh and Gupta, 2014; Mendoza, 2019; Salessi and Omar, 2019). On the other hand, perceived organizational support refers to the employees’ perception of how much their organization values and supports them (Eisenberger et al., 1986, 2002; Lynch et al., 1999). It encompasses the extent to which employees believe their organization cares about their wellbeing, recognizes their contributions, and provides resources and assistance when needed. In this context, job insecurity reflects employees’ concerns about the stability and continuity of their employment, including the fear of job loss or precariousness.

The existing literature reveals mixed findings regarding the relationship between perceived organizational support and job insecurity (Rosenblatt and Ruvio, 1996; Blackmore and Kuntz, 2011; Bohle et al., 2018; Zaki and Aini, 2022). Some studies suggest a negative correlation, indicating that higher levels of perceived organizational support are associated with lower levels of job insecurity. For instance, Rosenblatt and Ruvio (1996) concluded that perceived organizational support is a consequence of job insecurity. In other words, when employees feel secure in their jobs and perceive organizational support, they are less likely to experience job insecurity. On the contrary, Blackmore and Kuntz (2011) found that perceived organizational support is an antecedent to job insecurity. Their research suggests that when employees perceive low levels of support from their organization, they are more likely to feel insecure about their jobs. These conflicting findings suggest the possibility of a reciprocal or interactive relationship between perceived organizational support and job insecurity, where one variable can influence and be influenced by the other over time.

Similarly, the relationship between job involvement and job insecurity has yielded contradictory results in the literature (Probst, 2000; Peiro et al., 2012). Some studies suggest a negative correlation, indicating that lower levels of job involvement are associated with higher levels of job insecurity. For example, Peiro et al. (2012) found that employees who feel less involved in their work are more likely to experience higher levels of job insecurity. When individuals are less engaged and invested in their jobs, they may perceive a higher risk of job loss and instability. On the other hand, Probst (2000) presented a contrasting perspective, proposing a positive association between job involvement and job insecurity. According to this viewpoint, highly involved workers may experience heightened job insecurity because their work occupies a significant portion of their lives. They may fear losing their job or facing negative consequences if their job is threatened.

It is important to acknowledge that the relationship between perceived organizational support, job involvement, and job insecurity is complex and multifaceted, and it may vary depending on individual and contextual factors. Moreover, the existing literature on this specific relationship is limited and has produced divergent findings. However, these variables significantly influence employee attitudes, behaviors, and wellbeing. Understanding the dynamics between perceived organizational support, job involvement, and job insecurity is crucial for organizations to create a supportive and engaging work environment. Employees who perceive high levels of support from their organization are likelier to experience lower levels of job insecurity, leading to improved job satisfaction, organizational commitment, and performance (Peiro et al., 2012; Bohle et al., 2018). Conversely, employees who feel unsupported or lack involvement in their work may be more prone to job insecurity, which can have detrimental effects on their wellbeing and productivity. Hence, the second hypothesis developed as follows:

*H*2: Influence of job involvement and perceived organization support toward job insecurity.

*H*2a: Job involvement significantly predicts job insecurity.

*H*2b: Perceived organizational support significantly predicts job insecurity.

## 4 Job involvement, perceived organization support, and job insecurity with organizational commitment

The relationship between job involvement, perceived organizational support, job insecurity, and organizational commitment is crucial in understanding the dynamics of employee engagement and loyalty within an organization. Job involvement reflects employees’ engagement and dedication toward their work, while perceived organizational support captures employees’ perceptions of the organization’s support and care. Conversely, job insecurity represents the uncertainty and perceived risk regarding employment continuity. Organizational commitment signifies employees’ psychological attachment and loyalty to the organization. Understanding how these factors interrelate is essential for organizations to create a supportive work environment that fosters employee commitment and reduces turnover (Allen, 2001; Llobet and Fito, 2013; Wan Sulaiman et al., 2013; Gvpn et al., 2018; Ibrahim et al., 2018; Chow and Abdullah, 2019) This expanded analysis will explore the impact of job involvement, perceived organizational support, and job insecurity on organizational commitment, shedding light on the intricate connections between these variables and their implications for employee engagement and organizational success.

Traditionally, organizational commitment has been conceptualized and measured through its three dimensions: normative commitment, affective commitment, and continuance commitment (Meyer et al., 1993; Meyer and Allen, 1997). Normative commitment reflects an individual’s obligation to remain with an organization due to a sense of moral duty or loyalty. Affective commitment refers to an individual’s emotional attachment, identification, and organizational involvement. Continuance commitment encompasses an individual’s perception of the costs associated with leaving the organization, including factors such as the loss of benefits or investments made during their tenure with the organization. Together, these dimensions provide a comprehensive understanding of an individual’s commitment to the organization.

This research adopts a holistic approach to organizational commitment, considering it a latent variable encompassing all three dimensions. While recognizing the value of examining each dimension separately, our focus is on exploring the overall impact of organizational commitment on employee attitudes and behaviors. By taking a broader perspective, we aim to provide a comprehensive understanding of the role of organizational commitment in relation to job involvement, perceived organizational support, and job insecurity. By investigating the relationship between organizational commitment as a whole and its influences on job involvement and job insecurity, we believe that our study will contribute to the existing literature by providing a comprehensive understanding of how organizational commitment, in its entirety, affects employee attitudes and outcomes. Moreover, it allows for a practical and meaningful interpretation of organizational commitment, considering its multidimensional nature within the context of the examined variables.

Job involvement and perceived organizational support have a significant relationship that influences employees’ organizational commitment (Eisenberger et al., 1986; O’Driscoll and Randall, 1999; Rhoades and Eisenberger, 2002; Ngo et al., 2013; Pattnaik et al., 2020; Ridwan et al., 2020). Research has shown a positive association between job involvement and organizational commitment (O’Driscoll and Randall, 1999). When employees are highly involved in their jobs and feel a strong sense of dedication, it often translates into a higher level of commitment to the organization. Combining job involvement and perceived organizational support creates a synergistic effect on organizational commitment. Employees who are highly involved in their jobs and perceive strong organizational support reinforce their commitment and loyalty. They feel a sense of pride in their work and recognize the organization’s investment in their success, further deepening their commitment. This positive relationship between job involvement and perceived organizational support has important implications for employee engagement, satisfaction, and overall organizational performance. Organizations can promote employee commitment by fostering job involvement through meaningful work, autonomy, and growth opportunities while also creating a supportive work environment that demonstrates care, appreciation, and recognition for employees’ contributions (Eisenberger et al., 1986; O’Driscoll and Randall, 1999; Rhoades and Eisenberger, 2002; Ngo et al., 2013).

Perceived organizational support shapes employees’ organizational commitment. It encompasses the employees’ perception of the organization’s appreciation, recognition, care for their wellbeing, and overall support (Eisenberger et al., 1986; Rhoades and Eisenberger, 2002). Research consistently demonstrates a positive relationship between perceived organizational support and organizational commitment. Employees who perceive higher levels of support from their organization tend to exhibit higher levels of commitment to the organization (Ngo et al., 2013; Pattnaik et al., 2020). Perceived organizational support enhances organizational commitment by creating a positive work environment. When employees perceive that their organization cares about their wellbeing, provides opportunities for growth and development, and values their contributions, it increases their commitment and loyalty (Mowday et al., 1979). Research has shown that employees who perceive higher levels of organizational support are likelier to exhibit affective organizational commitment, which involves an emotional attachment and identification with the organization (Ngo et al., 2013; Suárez-Albanchez et al., 2022). When employees perceive higher levels of support from their organization, it creates a positive work environment, fosters emotional attachment, and enhances their commitment to the organization. Organizations can promote employee commitment by prioritizing and demonstrating care, recognition, and support for their wellbeing and contributions (Eisenberger et al., 1986; Rhoades and Eisenberger, 2002; Ngo et al., 2013). When employees feel appreciated, valued, and supported by their organization, they feel a sense of pride, leading to increased job satisfaction and willingness to go above and beyond their responsibilities.

Job insecurity has a significant impact on organizational commitment Research consistently shows a negative relationship between job insecurity and organizational commitment. Employees who experience higher levels of job insecurity tend to exhibit lower levels of commitment to their organization (Rosenblatt and Ruvio, 1996; Probst, 2000; De Witte and Naswall, 2003; Buitendach and De Witte, 2005; Adebayo, 2006; Sora et al., 2010; Ngo et al., 2013; Marques et al., 2014; Urbanaviciute et al., 2015; Vujičić et al., 2015; Frone, 2018; Stankeviciute et al., 2021). The uncertainty and perceived risk of job insecurity undermine employees’ sense of attachment, loyalty, and dedication to the organization. Job insecurity negatively affects organizational commitment through various mechanisms. Firstly, job insecurity creates a sense of instability and threat, leading to increased stress and anxiety among employees (Ashford et al., 1989). Secondly, job insecurity can lead to decreased job satisfaction and motivation. Employees who perceive a lack of job security may feel less invested in the organization and less willing to go above and beyond their responsibilities. Decreased motivation and satisfaction can diminish organizational commitment (Probst, 2000). Lastly, job insecurity can trigger active job search behavior as employees seek more secure employment elsewhere (Rosenblatt and Ruvio, 1996). This turnover intention and the desire for more stable job opportunities can significantly undermine employees’ commitment to the current organization. The uncertainty and fear associated with job insecurity contribute to decreased attachment, loyalty, and dedication to the organization. Organizations can foster a committed and engaged workforce by addressing job insecurity, which in turn increases productivity and overall organizational success.

Therefore, building on previous research findings, we developed the hypothesis as follows:

*H*3: Influence of job involvement, perceived organization support, and job insecurity toward organizational commitment.

*H*3a: Job involvement significantly predicts organizational commitment.

*H*3b: Perceived organizational support significantly predicts organizational commitment.

*H*3c: Job insecurity significantly predicts organizational commitment.

## 5 Mediating role of job insecurity

Over the past decades, job insecurity has been a subject of extensive research in organizational psychology, focusing on its impact on various aspects of employee wellbeing (Greenhalgh and Rosenblatt, 1984; De Witte, 1999). Past studies have primarily examined job insecurity as a mediator for psychological wellbeing and other job attitudes, such as burnout, work engagement, wellbeing, emotional intelligence, and job satisfaction among other variables (Cheng and Chan, 2008; De Cuyper et al., 2008; Baillien and De Witte, 2009; Safaria et al., 2011; Wan Sulaiman et al., 2013; Ouyang et al., 2014; Park and Ono, 2016). However, there is a noticeable gap in the research regarding job insecurity as a mediator within a comprehensive framework that combines job involvement, perceived organizational support, organizational commitment, and job insecurity.

While research has established the direct impact of job insecurity on employee wellbeing, limited attention has been given to its mediating role in other job attitudes crucial for organizational success and productivity (Vujičić et al., 2015; Kurnia and Widigdo, 2021). By examining job insecurity as a mediator, researchers can understand the underlying mechanisms through which these variables influence organizational commitment. Particularly in times of organizational change, employees grappling with job insecurity may find themselves doubting their connection to the organization, resulting in diminished levels of commitment. The uncertainty and fear associated with job insecurity can erode employees’ trust in the stability and longevity of their employment, reducing their commitment to the organization (Sverke et al., 2002). This is particularly relevant in today’s dynamic and uncertain work environments, where technological advancements, economic fluctuations, and industry disruptions contribute to heightened levels of job insecurity. When employees perceive their jobs as uncertain or unstable, they may question their ability to perform effectively and fulfill their organizational roles. These perceptions of job insecurity can lead to decreased organizational commitment, as employees may feel disconnected and disengaged from their work and the organization (Cheng and Chan, 2008).

When job involvement, perceived organizational support, and commitment are combined within a framework that considers job insecurity as a mediator, practitioners and organizational leaders can gain valuable insights into the processes through which these variables interact to impact employee performance. By considering job insecurity as a mediator, we can better understand how these variables collectively influence employee wellbeing and organizational commitment. Job insecurity may amplify or mitigate the effects of job involvement, perceived organizational support, and commitment on employee wellbeing. For example, high levels of job involvement and low levels of perceived organizational support may increase job insecurity, negatively impacting employee wellbeing and commitment to the organization. With that, this paper generated the hypothesis as follows:

*H*4: Job insecurity negatively mediates the relationship between job involvement and organizational commitment.

*H*5: Job insecurity negatively mediates the relationship between perceived organizational support and organizational commitment.

**Figure 1:**
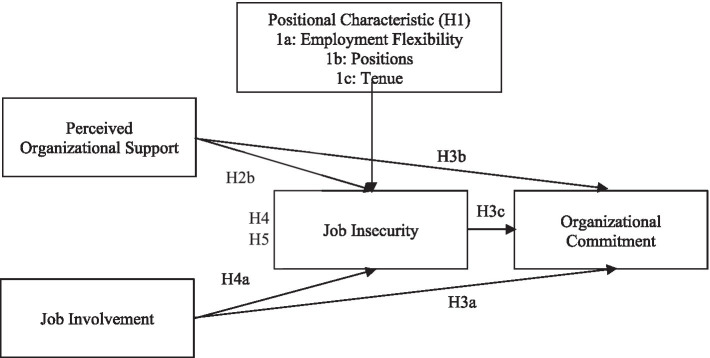
Relationship between variables.

Figure 1 shows the overall research model and path analysis.

## 6 Methodology

### 6.1 Procedure

Data were collected between May 2022 and August 2022 using an online questionnaire, sampling from 13,211 organizations listed in the Human Resources Development Corporation (HRDF) portal and organizations selected based on stratified random sampling for each industry. The strength of choosing stratified random sampling is that the population of working adults in the Private Sector in Malaysia is large, amounting to 8.475 million (DOSM, 2021), and the sample would need to break the population into a sub-group, followed by systematic sampling to obtain the data in a cost-effective and time-saving manner (Taherdoost, 2016). Email broadcast to 2,000 companies to request participation.

### 6.2 Materials

This study uses a structured questionnaire comprising one demographic questionnaire and four standardized measuring instruments. The questionnaires and their copy are shown as the following:

1. Screening Question.
2. Section 1, Informed Consent with Information.
3. Section 2, Demographic Questionnaire.
4. Section 3, which consisted of the job involvement questionnaire.
5. Section 4, which consisted of the survey of perceived organizational support.
6. Section 5, which consisted of the job insecurity questionnaire.
7. Section 6, which consisted of the three-component model of employee commitment.

#### 6.2.1 Screening question

The target population is the group of employed and salaried employees in Malaysia, particularly within the private sector. Hence, the screening question is set to ensure that the participants are eligible. The question asked is, “I am employed in Malaysia and work in the Private sector.” with the options given as “yes” and “no.” If the participant answers “no,” the survey will go straight to the “end of survey” page. This ensures that the survey reaches the targeted population, as negative responses during the screening will result in the termination of the survey.

#### 6.2.2 Informed consent

Informed consent is a form given to individuals who participate in the study or research to inform them of the content and purpose of the study (APA, 2017). The participants must be informed of the study’s purpose and processes and have the option to decide on their participation. Participation must be voluntary, and the participants can withdraw at any point in the study.

Participants Informed Consent with Information Sheet contains the basic information of the research and researcher, such as the purpose of the research, duration, procedures, and confidentiality information. It allows the participants to decide whether to participate in the research without any obligations. Consent will be obtained before they can proceed to participate in the survey.

#### 6.2.3 Demographic information

Participants’ demographic data, such as age, gender, race, relationship status, educational background, length of service, industry, position, and employment setting (days spent in the office), were asked at the beginning of the survey. The responses were measured according to a pre-coded nominal scale.

#### 6.2.4 Job involvement

The Job Involvement Questionnaire measures an individual’s psychological identification with job involvement (Kanungo, 1982). The instrument contains 10 items, administered using a Likert scale, ranging from (1) strongly disagree to (5) strongly agree. The instrument is reported with a one-dimensional variable of Cronbach Alpha coefficient ranging from 0.81 to 0.86 (Kanungo, 1982; Paterson and O’Driscoll, 1990; Hoole and Boshoff, 1998; Wegge et al., 2007). One sample question from the instrument is as follows: “I live, eat, and breathe my job.” The original scale encompassing 10 items was modified per the current research settings and generated six items with Cronbach alpha (reliability) of 0.852.

#### 6.2.5 Perceived organizational support

The survey of Perceived Organizational Support is used to measure the individual’s feeling of commitment by an organization toward its employees (Eisenberger et al., 1986; Eisenberger et al., 2002). There are a couple of versions available. The original version of the instrument consists of 36 items, measured using a 7-point Likert scale, from (0) = strongly disagree to (6) = strongly agree. A short version of 8 out of the 36 items measuring perceived organizational in various industries across different jobs, families, and functions was developed (Eisenberger et al., 1986; Eisenberger et al., 1997; Lynch et al., 1999; Shoss et al., 2013). One sample question from the instrument is as follows: “The organization really cares about my wellbeing.” In addition, it reported high internal reliability with Cronbach alpha of 0.90 (Eisenberger et al., 1997; Lynch et al., 1999). The original scale encompassing 8 items was modified per the current research settings and generated four items with Cronbach alpha (reliability) of 0.901.

#### 6.2.6 Job insecurity

The Job Insecurity Questionnaire (De Witte, 1999 as cited in De Cuyper and De Witte, 2005) is used to measure job insecurity in this study. There are a couple of versions available. A 4-item JIS was developed and translated into various languages (Vander Elst et al., 2014), which measures job insecurity and the threat or chances of losing the job. It has been conceptualized as the subjective perception and unsought chances of future job loss. One sample question from the instrument is as follows: “Chances are, I will soon lose my job.”The items are arranged along a Likert-type scale, varying from l = (strongly disagree) to 5 = (strongly agree) and reported to have high validity and can be considered a valid criterion scale to be used with Cronbach alpha coefficient reported between 0.85 and 0.88 (Van Hootegem et al., 2019) and this study reported having Cronbach alpha (reliability) of 0.842.

#### 6.2.7 Three-component model employee commitment survey

The TCM Employee Commitment Survey measures employees’ organizational commitment based on the three major components proposed by Allen and Meyer (1990): affective, continuance and normative commitment. Meyer et al. (1993) developed a revised version with 18 items, with 6 items per subscale. Both versions are self-administered with a 7-point Likert scale, ranging from (1) Strongly agree to (7) strongly disagree. Meyer et al. (1993) reported a Cronbach alpha coefficient of 0.87 for affective commitment, 0.79 for continuance commitment, and 0.73 for normative commitment. After modifying the original scale with eight items for the present study, six items were generated, with a Cronbach alpha of 0.843. One sample question from the instrument for affective commitment is as follows: “I really feel as if this organization’s problems are my own.”

## 7 Statistical analysis

The hypothesized model was tested using the IBM Statistical Package for Social Science (SPSS) for Windows Version 27.0 and structural equation modelling (SEM) using IBM SPSS Analysis of Moment Structures (AMOS) for Windows Version 28. A two-step approach was employed to analyze the efficacy of the proposed model. The first step involved testing a measurement model by examining one-dimensionality, convergent validity, construct validity, discriminant validity and reliability through confirmatory factor analysis (CFA), followed by examining the variables under investigation regarding their normality, kurtosis indices, and skewness. Also, means, standard deviations (SD), Cronbach’s alpha, and Pearson correlation coefficients between the key variables were calculated to examine the association between all study variables. In the next step, we tested a structural model by examining the relationships between variables through path estimates.

### 7.1 Handling common method variance

As Podaskoff et al. (2003) recommended, this research has implemented multiple strategies to mitigate the potential impact of common method variance (CMV) and social desirability bias. The study strongly emphasizes respondent anonymity, creating an environment where participants feel comfortable providing candid responses. Furthermore, the survey instrument utilized in this research has been meticulously designed to feature straightforward, unambiguous, and easily comprehensible items, reinforcing the notion that there are no inherently correct or incorrect answers. CMV is being addressed using Harman 1-factor analysis and Common Latent Factor analysis (CLF).

The Harman 1-factor analysis reported 25.485% shows CMV does not exist as it is less than the recommended threshold of 50%. The assumption is that if CMV existed, one factor would account for most of the variance. Results of CLF are presented in Table 1, which shows that common method bias is not a substantial concern in this research, with chi-square showing the same value with 1 df differences. With a non-significant common method bias test, there is no need to include the common method latent factor in the structural analysis, and it has shown that potential bias is not a concern moving forward.

**Table 1:** Common method variance analysis (CLF).

Model	Chi-square	Degree of freedom (df)
Original CFA model	474.197	159
CFA with common method factor	474.197	158

### 7.2 Measurement model

In the measurement model, we evaluated the reliability and validity of the latent variables. The internal consistency of the constructs was assessed using Cronbach’s alpha and composite reliability. Two important indicators, factor loadings and average variance extracted (AVE), were examined to establish convergent validity. The results presented in Table 2 show that Cronbach’s alpha values for all constructs ranged from 0.842 to 0.901, and the composite reliability values ranged from 0.839 to 0.909. These values surpass the threshold of 0.70 (Kline, 2005; Tabachnick and Fidell, 2007; Hair et al., 2010), indicating satisfactory internal reliability. The factor loadings of all latent variables ranged from 0.534 to 0.871, which is higher than the recommended value of 0.40 (Guadagnoli and Velicer, 1988; Kline, 2005), indicating adequate convergent validity. The AVE values for the constructs ranged from 0.472 to 0.695, exceeding the acceptable threshold of 0.40 when Cronbach’s alpha value is above 0.70 (Fornell and Larcker, 1981; Hair et al., 2010), indicating good convergent validity. Discriminant validity was established by comparing the square root of the AVEs of each construct with its corresponding correlations (Fornell and Larcker, 1981). The results in Table 3 indicate that the square root of the AVEs for each construct is greater than the inter-construct correlations, demonstrating good discriminant validity.

**Table 2:** Convergent validity and reliability.

Constructs	Items	Standardized factor loadings	Cronbach alpha	Composite reliability	AVE
Job involvement	6	0.534–0.765	0.852	0.850	0.489
Perceived organizational support	4	0.795–0.871	0.901	0.901	0.695
Job insecurity	4	0.598–0.815	0.842	0.840	0.571
Organizational commitment	6	0.546–0.871	0.843	0.840	0.472

**Table 3:** Discriminant validity.

		1	2	3	4
1	Job involvement	**0.699**			
2	Perceived organizational support	0.187	**0.833**		
3	Job insecurity	−0.122	−0.552	**0.756**	
4	Organizational commitment	0.433	0.557	−0.400	**0.687**

Bold diagonal values are the square root of AVE’s.

## 8 Results

The survey questionnaire was sent to the identified organizations and obtained a total of 554 respondents; 96 had missing data or did not complete the survey, resulting in 440 responses in the analysis, which yielded 79% qualified data (Table 4).

**Table 4:** Industry breakdown by economic activities.

Responses from online questionnaires	Number
Responses recorded	554
Disqualified (not employed in the private sector)	18
Incomplete/Missingdata	96
Qualified data	440

Table 5 provides a summary of the demographic details of the respondents. Gender analysis revealed that both male and female share 48.2% and 51.85, respectively, which mean the respondents included both male and female working in the relevant organizations. From the age perspective, it indicates that the majority (47.7%) of the respondents belong to the age group 35–44 category. In comparison, more than one-fifth (27.7) of the respondent’s age falls between 25 and 34, and a little less (19.5%) belongs to the 45–54 age category. A little (1.8%) belongs to the 55–64 and 60+ age categories. Only a small number (1.4%) of the respondents belong to the 18–24 age category. The respondents’ mean (M) age was 39.8; the results show a standard deviation (S.D.) of 0.87.

**Table 5:** Industry breakdown by economic activities.

Variable		Frequency	Percentage (%)	Cumulative %
Gender	Male	212	48.2	48.2
	Female	228	51.8	100.0
Age	18–24	6	1.4	1.4
	25–34	122	27.7	29.1
	35–44	210	47.7	76.8
	45–54	86	19.5	96.3
	55–60	8	1.8	98.2
	60 +	8	1.8	100.0
Race	Malay	60	13.6	13.6
	Chinese	358	81.4	95.0
	Indian	10	2.3	97.3
	Others	12	2.7	100.0
Position	Non-Executive	44	10.0	10.0
	Executive	132	30.0	40.0
	Middle Management	134	30.5	70.5
	Management	130	29.5	100.0
Tenure	< 2 years	128	29.1	29.1
	> 2–5 < years	104	23.6	52.7
	> 5–10 < years	104	23.6	76.4
	10 & above	104	23.6	100.0
Days Required to be in Office (Employment Flexibility)	None	50	11.4	11.4
	1 day >	6	1.4	12.7
	1–2 days >	28	6.4	19.1
	2–3 days >	38	8.6	27.7
	3 days & above	318	72.3	100.0

Concerning the race or ethnicity of the respondents, the results show that the majority (81.4%) of the respondents were Chinese, while less than one-fifth (13.6%) of the respondents were Malay. The results also reveal that a few (2.3%) were Indians, and only (2.7%) were from other races or ethnicities. According to DOSM, Current Population Estimates, Malaysia, 2022, divisions by ethics are as follows: Malay (69.6%); Chinese (22.8%); Indian (6.7%); and Others (0.7%); however, Free Malaysia Today (2022) revealed that 90% of the civil servant are Malays. Hence, this could contribute to the ethnicity breakdown of the respondent.

Employment flexibility analysis shows that most (72.3%) respondents must present at least 3 days physically and above a week, while as little as (11.4%) are not required to do so. There are significantly fewer respondents who are practicing hybrid working environments. Results show that only (8.6%) of them have to report to the office physically between 2 and 3 days, (6.4%) have to present physically for 1–2 days, and (1.4%) have to come 1 day in a week physically. The M days were 4.29, and S.D. was reported at 1.33. In summary, only 16.4% of the respondents work in organizations that practice hybrid working environments despite the pandemic largely changing the way forward.

Results revealed that for position level, most respondents range from Executive to Management level, with 30% at the executive level, 30.5% in middle management and 29.5% at the management level. Out of it, only 10% of the respondents are from the non-executive level. Language could be the potential hindrance or limitation of getting the non-executive level to participate in the survey. Results indicated most respondents served the current organization for less than 2 years (29.1), and the same number of respondents (23.6%) served their organization between two to less than 5 years, five to less than 10 years, and 10 years and above. The M of the years working is 2.41, and the SD is 1.14.

**Figure 2:**
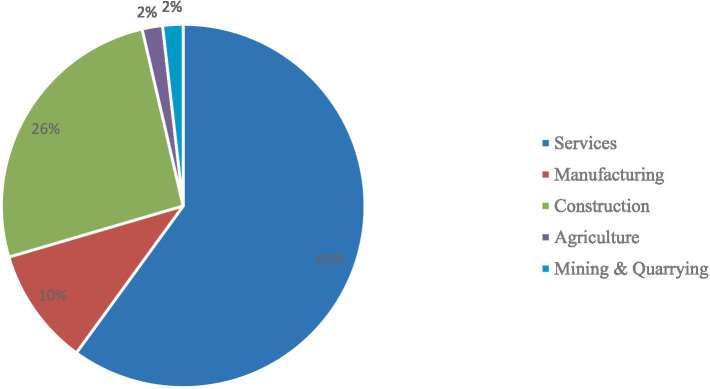
Industry distribution by respondents.

Figure 2 indicates industry distribution by respondents; the result shows that most (60%) of the respondents work in the services sector and (25.9%) in the construction sector. Fewer than one-fifth (10.5%) of the respondents work in the manufacturing industry, and only (1.8%) of the respondents work in mining and quarrying, respectively. The distribution of the industry is close to the actual distribution as published by DOSM (2021) as shown in Table 6. Results suggested that the findings have the potential for generalizability to employees across the private sector in Malaysia.

**Table 6:** Mean, standard deviation, and Pearson correlations analysis.

Industry	Respondents	Distribution (%)	Ideal distribution	Distribution by population (%)	Variance
Services	264	60%	228	52%	−8%
Manufacturing	46	10%	117	27%	16%
Construction	114	26%	66	15%	−11%
Agriculture	8	2%	25	6%	4%
Mining and quarrying	8	2%	4	1%	−1%

Table 7 displays the study variables’ means (M), standard deviations (S.D.), and correlations.

**Table 7:** Mean, standard deviation, and Pearson correlations analysis.

Variables	M	SD	1	2	3
1. Perceived organizational support	3.66	1.26			
2. Job insecurity	2.22	0.81	−0.52**		
3. Job involvement	3.21	0.59	0.19**	−0.14**	
4. Organizational commitment (OC)	4.16	0.87	0.47**	−0.34**	0.41**

**Significant (2-tailed) < 0.001.

The results of the ANOVA indicated significant differences between job insecurity and employment flexibility, job positions, and tenure (Table 8). Specifically, there is a significant difference in employment flexibility among employees, *F*(4, 435) = 3.515, *p* = 0.008; the effect size, calculated as eta squared (η^2^), was 0.031. Furthermore, there is a significant difference in job positions, *F*(3, 436) = 7.371, *p* < 0.001; the effect size, calculated as eta squared (η^2^), was 0.048. Lastly, there is a significant difference in tenure, *F*(3, 436) = 4.159, *p* = 0.006; the effect size, calculated as eta squared (η^2^), was 0.028. These results suggest that positional characteristics may influence job insecurity differently, depending on the specific characteristic being considered, and effect size indicated that there may be limitation in practical applications.

**Table 8:** ANOVA between job insecurity and positional characteristic.

Dependent variable	Model	S.S.	df	M.S.	F	Sig.	η^2^
Employment flexibility	Between groups	8.918	4	2.230	3.515	0.008	0.031
	Within groups	275.956	435	0.634			
	Total	284.874	439				
Job positions	Between groups	13.751	3	4.584	7.371	<0.001	0.048
	Within groups	271.123	436	0.622			
	Total	284.874	439				
Tenure	Between groups	7.925	3	2.642	4.159	0.006	0.028
	Within groups	276.950	436	0.635			
	Total	284.874	439				

SS, Sum of Squares; df, degrees of freedom MS, mean square; η^2^, Eta squared. The mean difference is significant at the 0.05 level.

A Tukey’s Honestly Significant Difference (HSD) test for multiple comparisons was applied to find the comparison between job insecurity and positional characteristics: employment flexibility (Table 9), positions (Table 10), and tenure (Table 11). The table shows the mean differences in job insecurity between different employment flexibility levels, their significance level (Sig.), and corresponding confidence intervals (Lower Bound and Upper Bound). Significant mean differences (*p* ≤ 0.05) are indicated with an asterisk (*).

**Table 9:** Mean differences between job insecurity and employment flexibility.

Employment flexibility		Mean difference	Sig.	95% confidence interval	
				Lower bound	Upper bound
Not at all	1 day	0.217	0.970	−0.7260	1.1593
	1–2 days	−0.521	0.046*	−1.0364	−0.0064
	2–3 days	0.089	0.985	−0.3801	0.5590
	3 days and above	−0.213	0.402	−0.5445	0.1193
1 day	1–2 days	−0.738	0.240	−1.7196	0.2434
	2–3 days	−0.127	0.996	−1.0856	0.8313
	3 days and above	−0.429	0.687	−1.3283	0.4698
1–2 days	2–3 days	0.611	0.019*	0.0675	1.1543
	3 days and above	−0.309	0.284	−0.1212	0.7389
2–3 days	3 days and above	−0.302	0.178	0.0675	1.1543

*p ≤ 0.05 significant mean differences.

**Table 10:** Mean differences between job insecurity and job positions.

Employment flexibility		Mean difference	Sig.	95% confidence interval	
				Lower bound	Upper bound
Non-executive level	Executive level	0.102	0.879	−0.2517	0.4563
	Middle management	0.115	0.837	−0.2387	0.4680
	Management level	0.474	0.003**	0.1194	0.8288
Executive level	Middle management	0.012	0.999	−0.2370	0.2618
	Management level	0.372	0.001***	0.1206	0.6231
Middle management	Management level	0.359	0.001***	0.1091	0.6098

**p ≤ 0.01, ***p ≤ 0.001 significant mean differences.

**Table 11:** Mean differences between job insecurity and tenure.

Tenure of service		Mean difference	Sig.	95% confidence interval	
				Lower bound	Upper bound
Below 2 years	2–5 years	−0.370	0.003^*^	−0.6415	−0.0988
	> 5–10 years	−0.144	0.518	−0.4156	0.1271
	> 10 years and above	−0.178	0.330	−0.4492	0.0935
>2–5 years	> 5–10 years	0.226	0.173	−0.0591	0.5110
	10 years and above	0.192	0.304	−0.0927	0.4773
> 5–10 years	10 years and above	−0.034	0.990	−0.3187	0.2514

*p ≤ 0.05 significant mean differences.

Table 9 presents the mean differences between job insecurity and employment flexibility. Overall, there is a significant mean difference in job insecurity between employees with different levels of employment flexibility. Specifically, employees with 1–2 days of employment flexibility experience significantly higher job insecurity than those without employment flexibility (mean difference = − 0.521, *p* = 0.046). The only other significant mean difference is between employees with 1–2 days and 2–3 days of employment flexibility, with the latter group experiencing significantly lower job insecurity (mean difference = 0.611, *p* = 0.019). However, there was no statistically significant difference among the means of the remaining groups of job insecurity and employment flexibility recorded.

Table 10 presents the mean differences between job insecurity and job positions in comparing non-executive, executive, middle management, and management positions. There is a statistically significant difference in job insecurity management level with all other, non-executive (mean Difference = 0.474, *p* = 0.003), executive-level (mean Difference = 0.372, *p* = 0.001), middle management (mean difference = 0.359, *p* = 0.001). No other significant difference between the remaining groups was recorded because the value of p of these groups was greater than 0.05.

Table 11 presents the mean differences between job insecurity and tenure of service for different tenure categories. According to the results, the mean difference in job insecurity is highest for employees with 2–5 years of tenure. For employees with less than 2 years of tenure, there is a significant mean difference in job insecurity compared to those with 2–5 years of tenure, with a mean difference of −0.370 (*p* ≤ 0.05). There is no significant difference in job insecurity with the rest of the group. Overall, the results suggest that job insecurity is highest among employees with 2–5 years of tenure and less than 2 years reported feeling secure.

All three hypotheses of H1a, H1b, and H1c were supported. These results suggest that positional characteristics may influence job insecurity differently, depending on the specific characteristic being considered. These findings underscore the importance of considering employees’ positional characteristics when examining job insecurity.

The results of the path analysis are presented in Table 12. Hypotheses show that job involvement is negatively associated but does not significantly predict job insecurity (*β* = −0.022, *p* > 0.05). Perceived organizational support negatively and significantly predicts job insecurity (*β* = −0.241, *p* < 0.05). Results show that all three variables significantly predict organizational commitment with job involvement positively (*β* = 0.698, *p* < 0.05), perceived organizational support positively (*β* = 0.416, *p* < 0.05), and job insecurity negatively (*β* = −0.275, *p* < 0.05).

**Table 12:** Regression weight.

Paths	Estimate	S.E.	C.R.	*p*-value	Hypothesis
JIS <--- JI	−0.018	0.046	−0.390	0.696	H2a—not supported
JIS <--- POS	−0.241	0.028	−8.677	< 0.001	H2b—supported
OC <--- JI	0.698	0.100	6.961	< 0.001	H3a—supported
OC <--- POS	0.416	0.057	7.348	< 0.001	H3b—supported
OC <--- JIS	−0.274	0.129	−2.120	0.034	H3c—supported

JIS, Job Insecurity; JI, Job Involvement; POS, Perceived Organizational Support; OC, Organizational Commitment; S.E., Standard Error; C.R., Critical Ratio.

H2a predicts that job involvement significantly predicts job insecurity. The findings revealed that job involvement has a non-significant inverse effect on job insecurity (*p* > 0.05). Hence, H3a was not supported by the given results.

H2b assumes that perceived organizational support significantly predicts job insecurity. The data findings also revealed that perceived organizational support has significantly predicted job insecurity. Hence, H3b was supported by the data. Results show that one unit change in perceived organizational support will significantly bring a 24.1% inverse change in job insecurity.

All three hypotheses under H3 were supported. Job involvement (H3a), perceived organizational support (H3b), and job insecurity (H3c) significantly predict Organizational commitment. A change in the unit of job involvement and perceived organizational support will increase organizational commitment by 69.8 and 41.6%, respectively. Job insecurity negatively predicts organizational commitment; a unit increase will decrease by 27.4% in organizational commitment.

**Figure 3:**
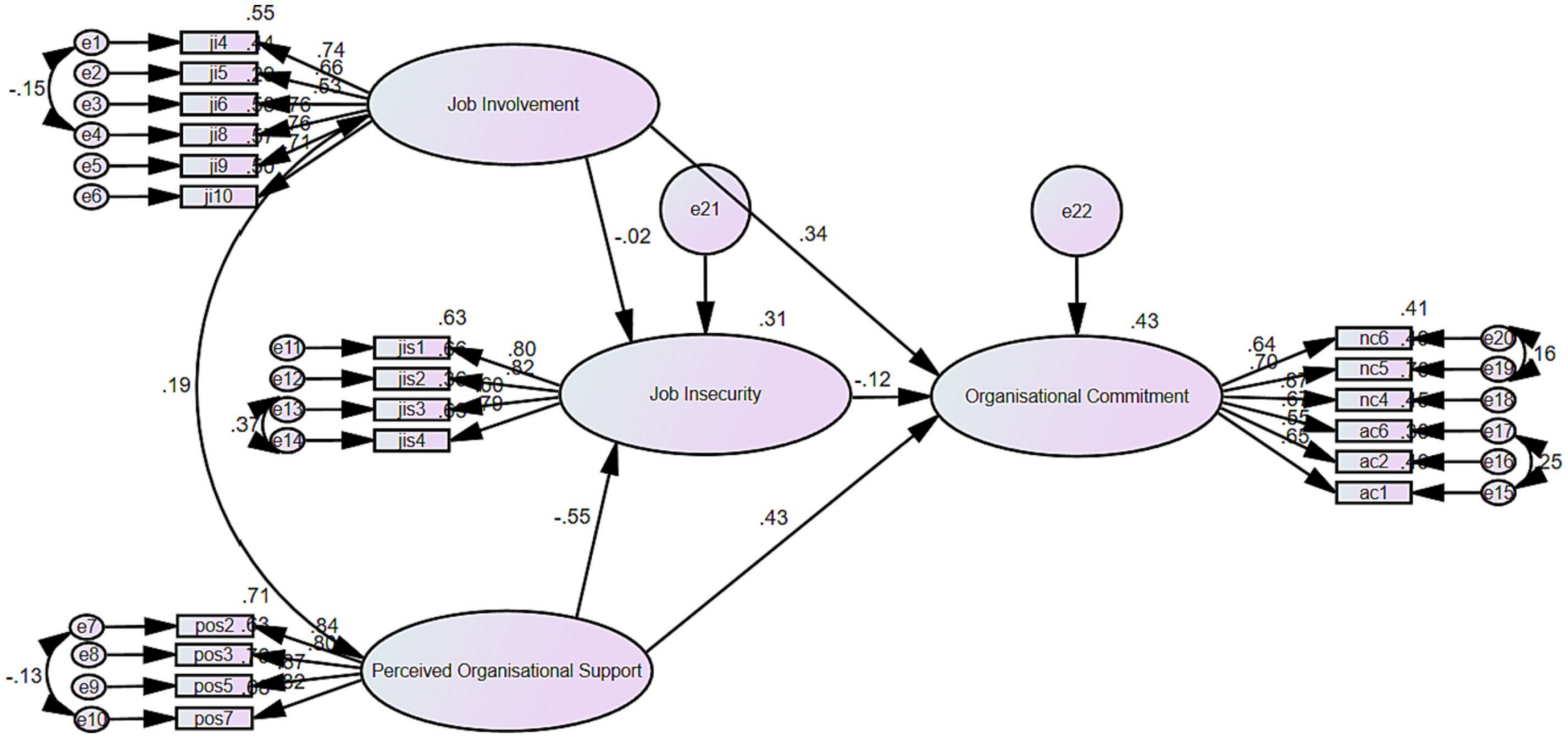
Structure equation model (SEM).

Mediation analysis was performed to assess the mediating role of job insecurity in the relationship between job involvement and organizational commitment (H4) and perceived organizational support and organizational commitment (H5), as shown in Figure 3. To assess the model fit, the researchers employed several model fit indices: relative/normed chi-square (CMIN/DF), the goodness of fit index (GFI), comparative fit index (CFI), Tucker-Lewis Index (TLI) and root mean square error of approximation (RMSEA). The acceptable values recommended for these estimates are greater than 0.90 for GFI (Hu and Bentler, 1998; Kline, 2005), greater than 0.90 for CFI (West et al., 2012), greater than 0.90 for TLI (Bentler and Bonett, 1980), less than 0.08 for RMSEA (MacCallum et al., 1996) and less than 3 for CMIN/DF (Marsh and Hocevar, 1985; Kline, 2005). As reported in Table 13, the model’s overall fit was reasonable on all indices (CMIN/DF = 2.983, GFI = 0.907, CFI = 0.928, TLI = 0.914 and RMSEA = 0.067).

**Table 13:** Model fit indicates for pooled construct.

CFA model	*p*-value	RMSEA	GFI	CFI	TLI	CMIN/df
1	0.000	0.067	0.907	0.928	0.914	2.982

The mediation analysis treats job involvement and perceived organizational support as independent variables, Organization commitment as dependent variables and Job insecurity as the mediator. The mediation analysis is based on the analysis of indirect effects based on the guideline by Baron and Kenny (1986) classical approach; mediation analysis is performed by using the direct and indirect effects based on bootstrap procedures (2,000 samples) and bias-corrected bootstrap confidence interval (95%).

The study assessed the mediating role of job insecurity on the relationship between job involvement and organizational commitment. Table 14 revealed a non-significant indirect effect of the impact of perceived organizational support on organizational commitment (*β* = 0.003, *p* = 0.489). Although the direct effect of perceived organizational support on job insecurity in the presence of the mediator was significant (*β* = 0.698, *p* = 0.001), there is a lack of evidence to conclude the presence or absence of a mediation effect.

**Table 14:** Mediation analysis summary.

Relationship	Direct effect		Indirect effect		Conclusion
	Beta (95% CI)	*p*-value	Beta (95% CI)	*p*-value	
Job involvement - > job insecurity - > organizational commitment	0.698 (0.495 to 0.918)	0.001	0.003 (−0.014 to 0.038)	0.489	Not supported
Perceived organizational support - > job insecurity - > organizational commitment	0.416 (0.318 to 0.531)	0.001	0.064 (0.011 to 0.122)	0.050	Partial mediation

CI, Confidence Interval.

The study also assessed the mediating role of job insecurity on the relationship between perceived organizational support and organizational commitment. The results revealed that a significant indirect effect of job involvement on organizational commitment was positive and significant (*β* = 0.416, *p* = 0.001). Furthermore, the direct effect of job involvement on job insecurity in the presence of the mediator was also significant (*β* = 0.064, *p* = 0.05). Hence, results show that job insecurity partially mediated the relationship between perceived organizational support and perceived organizational support.

## 9 Discussion

The study aimed to examine how work attitudes and perceived relationships affect organizational commitment among employees in the private sector in Malaysia, with job insecurity as a mediator. In addition, this study explored how employment flexibility, job position and years of service affect job insecurity. Table 15 summarizes the analysis results based on the hypotheses presented.

**Table 15:** Summary of result findings.

No	Hypotheses statement	Analysis	Sig.	Finding
H1	Differences in job insecurity based on positional characteristics (the mean difference is significant at the 0.05 level)	One way analysis of variance (ANOVA)		
1a	Job insecurity differs based on employment flexibility.		0.008	Accepted
1b	Job insecurity differs based on job positions.		<0.001	Accepted
1c	Job insecurity differs based on tenure.		0.006	Accepted
H2	Influence of job involvement and perceived organization support toward job insecurity	Regression analysis		
2a	Job involvement significantly predicts job insecurity.		0.696	Not accepted
2b	Perceived organizational support significantly predicts job insecurity.		<0.001	Accepted
H3	Influence of job involvement and perceived organization support toward organizational commitment.			
3a	Job involvement significantly predicts organizational commitment.		<0.001	Accepted
3b	Perceived organizational support significantly predicts organizational commitment.		<0.001	Accepted
3c	Job insecurity significantly predicts organizational commitment.		0.034	Accepted
H4	Job insecurity negatively mediates the relationship between job involvement and organizational commitment.	Mediation analysis	0.489	Not accepted
H5	Job insecurity negatively mediates the relationship between perceived organizational support and organizational commitment.		0.050	Accepted (partially mediated)

### 9.1 Differences in job insecurity based on positional characteristics

ANOVA was used to compare the means of the groups to determine differences in job insecurity based on positional characteristics of employment flexibility, job positions and tenure of service. Hypotheses 1a (employment flexibility), 1b (job positions), and 1c (tenure) are supported and show a significant relationship with job insecurity. Among the three, job positions were the strongest correlated with job insecurity, showing the strongest *F* value. Contradictory to the research conducted, positional characteristics have proven to influence job insecurity significantly (Bose and Biswas, 2019).

#### 9.1.1 Employment flexibility

First, the mean difference between job insecurity for employees with no employment flexibility and those with 1–2 days of flexibility is statistically significant, meaning that employees with at least some control over their work schedules experience less job insecurity than those without no control. Second, the mean Difference between job insecurity for employees with 1–2 days of flexibility and those with 2–3 days of flexibility is also statistically significant but in the opposite direction. It is suggested that there may be a “sweet spot” for employment flexibility, beyond which additional flexibility may not have as much of an impact on reducing job insecurity. Finally, the mean difference between job insecurity for employees with 2–3 days of flexibility and those with 3 days or more of flexibility is not statistically significant. Results also suggest that there is a point at which additional employment flexibility does not have a significant impact on reducing job insecurity.

The significant relationship between job insecurity and employment flexibility suggests that having some degree of control over one’s work schedule can positively impact an employee’s perceived job security. This finding is consistent with research showing that employment flexibility, such as flexible work hours or telecommuting, can give employees a sense of autonomy and control over their work, reducing stress and improving wellbeing. The fact that the mean difference in job insecurity is only significant between employees with no employment flexibility and those with 1–2 days of flexibility, and not between employees with higher levels of flexibility, maybe because the benefits of employment flexibility may be maximized at a certain level, beyond which further increases in flexibility may not provide additional benefits.

The impact of these results is that employers may want to consider providing employees with at least some degree of employment flexibility to improve their sense of job security and wellbeing. Human resource practices could offer flexible work schedules, telecommuting options, or other forms of flexibility that allow employees more control over their work. Doing so may improve employee satisfaction, wellbeing, and organizational outcomes such as productivity and retention. Factors beyond employment flexibility may also play a role in determining job insecurity, such as job stability, pay, and career advancement opportunities.

#### 9.1.2 Positions

The findings suggest that job insecurity varies significantly based on job position. Specifically, management-level positions experience higher job insecurity than non-executive, executive-level positions and middle-management. It could be due to higher job demands and expectations, greater exposure to organizational changes and restructuring, and a greater sense of responsibility for the organization’s success. The implications of these findings are important for both employees and employers. Employees in management-level positions may experience greater stress and anxiety levels due to job insecurity, which could negatively impact their wellbeing and job performance.

Furthermore, employers may need to consider the potential impact of job insecurity on employee retention and recruitment efforts. High levels of job insecurity could lead to higher turnover rates and difficulty in attracting top talent to management-level positions. Employers may need to prioritize creating a stable and supportive work environment to retain and attract talented employees. Employers may need to consider providing additional support and resources to these employees, such as counseling services, training and development opportunities, and clear communication about organizational changes.

#### 9.1.3 Tenure

The results show that the organization’s focus is addressing job insecurity among employees who have been with the company for 2–5 years. These employees are reported to experience the highest levels of job insecurity compared to others. On the other hand, newly recruited employees experience the lowest job insecurity; it could be due to both the organization and the employee being aware that new joiners are taking time to familiarize themselves with the organization, and both parties are assessing the suitability for long-term relationships.

Organizations can use this information to identify employees at higher risk of experiencing job insecurity and take proactive steps to address their concerns. To effectively tackle this issue, organizations can implement targeted interventions to reduce job insecurity and increase job satisfaction among employees within this tenure range. Interventions of this nature can encompass strategies such as open communication and transparency, which enhance communication channels and provide employees with clear information about organizational objectives, changes, and performance. Transparent communication plays a pivotal role in keeping employees well-informed, thereby mitigating uncertainty and reducing levels of job insecurity. Another effective intervention involves offering career development opportunities, including specialized training programs, mentoring initiatives, and tailored career development pathways designed specifically for employees with 2–5 years of tenure. By investing in their professional growth, organizations demonstrate a commitment to their employees’ future, which can help alleviate job insecurity. With that, organizations can help create a more stable and secure work environment, leading to higher employee engagement, retention, and commitment.

These results suggest that employment flexibility, job position, and tenure are all important factors contributing to employees’ job insecurity levels. Employees with less employment flexibility, lower job positions, and shorter tenure are likely to experience higher levels of job insecurity than those with more control over their work schedules, higher positions, and longer tenure.

### 9.2 Regression analysis

Hypotheses 2 and 3 examine the influence of job involvement and perceived organizational support on job insecurity and organizational commitment. Contrary to the hypothesis (H2a), the results did not support a significant relationship between job involvement and job insecurity. This finding suggests that an individual’s level of job involvement does not directly impact their perception of job insecurity. However, our hypothesis (2b) regarding the relationship between perceived organizational support and job insecurity was supported. The result indicates that higher levels of perceived organizational support are associated with lower levels of job insecurity. This finding suggests that employees feel more secure when they perceive greater organizational support. Organizations should prioritize creating a supportive work environment to alleviate employees’ concerns about job insecurity.

Hypothesis 3 examines the influence of job involvement (H3a), perceived organizational support (H3b), and job insecurity (H3c) on organizational commitment. Notably, job involvement exhibits a statistically significant positive relationship with organizational commitment. This outcome suggests that employees who display greater engagement with their work tend to demonstrate higher levels of commitment to their organization. This finding resonates with prior research, such as the work of O’Driscoll and Randall (1999), which underscores the enduring relevance of job involvement in cultivating organizational commitment. Despite the passage of more than three decades since the publication of that study, our results affirm its continued applicability in contemporary contexts.

Additionally, perceived organizational support was positively associated with organizational commitment. The regression analysis suggests that when employees perceive higher levels of support from their organization, they are more likely to be committed to it. This finding emphasizes the crucial role of organizational support in fostering employees’ commitment and loyalty. Furthermore, the analysis revealed a significant negative relationship between job insecurity and organizational commitment (H3c). The results indicate that higher levels of job insecurity are associated with lower levels of organizational commitment. This finding implies that job insecurity can undermine employees’ organizational commitment. Organizations should address and mitigate job insecurity to promote a stronger sense of commitment among their employees.

Overall, the results highlight the significance of perceived organizational support influencing job insecurity and organizational commitment. Creating a supportive work environment can help reduce employees’ perception of job insecurity and enhance their commitment to the organization. Although job involvement did not directly predict job insecurity, it significantly predicted organizational commitment. Hence, it is important to foster employee job involvement to enhance their commitment to the organization. Organizations should focus on creating a work environment that encourages employee engagement, autonomy, and opportunities for meaningful work through task variety, clear goal setting, recognition of employees’ contributions, and fostering a sense of purpose in work.

The significant negative relationship between job insecurity and organizational commitment suggests that job insecurity negatively affects organizational commitment; organizations should proactively address and alleviate job insecurity concerns among employees. It can be achieved through open and honest communication about the organization’s stability, providing opportunities for skill development and training, and implementing policies that promote job security and stability.

### 9.3 Mediation analysis

The findings of this study shed light on the complex relationship between job involvement, perceived organizational support, job insecurity, and organizational commitment. The results provide insights into how these factors interplay and influence employees’ commitment to their organizations. It confirms the relationship between job involvement and organizational commitment and indicates a positive direct effect. It implies that employees who are highly involved in their work are more likely to exhibit a stronger commitment to their organization. Job involvement reflects the extent to which individuals are engaged, absorbed, and dedicated to their work tasks. Consequently, organizations should foster an environment that encourages and supports employees’ active engagement and investment in their job roles, as it positively contributes to their commitment to the organization.

Interestingly, the analysis reveals that job insecurity does not significantly mediate the relationship between job involvement and organizational commitment. It is suggested that while job involvement directly influences organizational commitment, employees’ perceived job insecurity does not significantly impact this relationship. Organizations should not overlook the importance of fostering job involvement even in the presence of job insecurity concerns. By encouraging employees’ active participation and engagement, organizations can promote a sense of belonging and dedication that goes beyond the negative effects of job insecurity.

The finding aligns with prior research demonstrating that employees who perceive higher organizational support are likelier to exhibit stronger organizational commitment (Eisenberger et al., 1986; Rhoades and Eisenberger, 2002; Ngo et al., 2013; Pattnaik et al., 2020). Perceived organizational support encompasses employees’ beliefs about how much the organization values their contributions, cares about their wellbeing, and provides resources and assistance when needed. Such support fosters a sense of reciprocity and loyalty among employees, leading to higher levels of commitment to the organization.

Moreover, results reveal that job insecurity partially mediates the relationship between perceived organizational support and organizational commitment. The indirect effect of perceived organizational support on organizational commitment through job insecurity is significant. It suggests that when employees perceive lower levels of organizational support, it increases their level of job insecurity, which, in turn, influences their commitment to the organization. Organizations should, therefore, focus on creating a supportive work environment that actively addresses employees’ concerns and provides them with the necessary resources, feedback, and opportunities for growth. By reducing job insecurity, organizations can help mitigate its negative impact on employees’ commitment levels.

The results highlight the importance of job involvement and perceived organizational support in shaping organizational commitment. Job involvement directly contributes to organizational commitment, while perceived organizational support influences commitment directly and indirectly through its effect on job insecurity. Organizations should prioritize strategies that enhance job involvement and perceived organizational support while addressing job insecurity concerns to foster higher levels of organizational commitment among their employees. Organizations can create a positive work environment that promotes employee wellbeing, satisfaction, and commitment by understanding and acting upon these factors.

## 10 Theoretical, empirical, and practical contributions

The significance of this research lies in its contribution to increasing productivity and sustainability, retaining talent, and reducing voluntary attrition within organizations. This study contributes significantly to the view of theoretical contribution by examining the relationship between job involvement, perceived organizational support, job insecurity, and organizational commitment and incorporating job insecurity as a mediator. Integrating these variables into one research framework fills a gap in the existing literature, as previous studies have often examined these variables in isolation or focused on different aspects of the relationships (Shoss, 2017; Hngoi et al., 2023). Considering the interplay among the variables, this study provides a more comprehensive understanding of the factors influencing organizational commitment. It creates a linkage between the positional characteristics and provides clarity on the element that affects job insecurity.

The empirical findings of this study hold significant practical implications for organizations, particularly within the Human Resources or Talent Management divisions. By studying the antecedents of job insecurity, such as job involvement and perceived organizational support, and their relationship with organizational commitment, this research provides valuable insights for practitioners (Shoss, 2017; Saeed et al., 2023). It offers a more accurate and reliable source of information for designing effective talent retention strategies and allocating resources to increase employee productivity. The study also analyses job insecurity based on demographic differences, particularly organizational conditions. This analysis enables organizations to identify specific areas of concern and tailor policies, compensation and benefits strategies, and other interventions to address the needs of different employee groups. Organizations can develop strategies to mitigate insecurity and enhance job satisfaction and commitment by understanding the impact of employment flexibility on job insecurity. Organizations can indirectly boost productivity and improve employee morale by addressing job insecurity and low organizational commitment. This research empowers practitioners to make informed decisions and allocate resources effectively, reducing employee turnover costs and stress-related medical expenses. The saved resources can then be redirected toward initiatives to increase employee productivity and motivation.

In conclusion, this research makes significant theoretical contributions by examining the relationships between the variables and incorporating the mediating role of job insecurity. The study expands existing theoretical frameworks and provides a more comprehensive understanding of the factors influencing organizational commitment. Moreover, this research’s empirical and practical contributions enable practitioners to develop targeted strategies to enhance employee retention, productivity, and wellbeing. Organizations can foster a supportive work environment and improve overall performance by addressing job involvement, perceived organizational support, and job insecurity (Sverke et al., 2019; Pattnaik et al., 2020; Shao et al., 2022; Junça Silva and Lopes, 2023). Table 16 summarizes the study’s research gaps, findings, contributions, and implications.

**Table 16:** Summary of research gaps, research findings, and research contributions.

Research gap	Research finding	Research contribution	Research implications
A lack of research examines the relationship between JIS and positional characteristics.	JIS differs based on positional characteristics. *Employment flexibility* The significant mean Difference between not at all with 1–2 days and 1–2 days with 2–3 days. A “sweet spot” of flexibility, job insecurity was reported at the highest mean on medium flexibility compared to the rest, even with no flexibility. *Positions* Employees at the management level experience the highest level of job insecurity, which may be due to the higher job demands and expectations, greater exposure to organizational changes and restructuring, and a greater sense of responsibility for the organization’s success. *Tenure* The highest mean score for job insecurity was reported for employees serving 2–5 years and the lowest for less than 2 years. Employees have been serving for more than 2 years, settling in within organizations and starting to look at more opportunities and development.	*Theoretical contributions*: The research integrates and expands existing theoretical perspectives by linking JI, POS, JIS, and OC into a single research framework, filling a gap in the existing literature. It enhances understanding of stress development phenomena and their relationship with organizational dynamics, particularly the impact of JIS on OC.	*Research implications*: The findings have practical implications for H.R. or T.M. divisions by offering valuable information for designing effective talent retention strategies and resource allocation to enhance employee productivity.
A lack of research examines the relationship between JI POS, JIS and OC.	*A significant prediction between the variables*: POS negatively predicts JIS. JI and POS positively predict OC. JIS negatively predicts OC.	*Empirical contributions*: The study provides empirical evidence on the relationships between variables and examines the mediating role of JIS in the relationship between these variables. The research analyses job insecurity based on demographic differences and organizational conditions, providing practical insights for organizations.	It offers information for tailoring policies, compensation and benefits strategies, and interventions to address the needs of different employee groups based on demographic differences and organizational conditions. Practitioners can make informed decisions and allocate resources effectively, reducing employee turnover costs and stress-related medical expenses.
A dearth of research examines the mediating effect of JIS in the relationship between JI, POS, and OC.	*JIS partially mediates the relationship between POS and OC.* It suggests that when employees perceive lower levels of POS, it increases their level of JIS, which, in turn, influences their commitment to the organization.		

JI, Job Involvement; POS, perceived organizational support; JIS, job insecurity; OC, Organizational commitment; H.R., human resources; T.M., talent management.

### 10.1 Future research

Building upon the insights gained from this research, several promising directions for future investigations emerge. First, future research could benefit from longitudinal designs to investigate the temporal dynamics of the relationships between job involvement, perceived organizational support, job insecurity, and organizational commitment. Longitudinal studies would provide a more comprehensive understanding of these variables’ causal links and effects over time. Second, a mixed-methods approach combining quantitative data with qualitative methods, such as interviews or focus groups, could offer deeper insights into the experiences and perceptions of employees. This approach would provide a more holistic understanding of the phenomena under investigation.

Third, future studies could explore additional mediating and moderating variables that may influence the relationships among the variables. For instance, the role of psychological wellbeing, organizational culture, or leadership styles could be examined as potential mediators or moderators to enhance our understanding of the complex interplay among these variables. Fourth, comparative studies across different industries, sectors, or countries would provide valuable insights into the contextual factors influencing the relationships between job involvement, perceived organizational support, job insecurity, and organizational commitment. By comparing the relationships across diverse contexts, researchers can uncover the boundary conditions and cultural influences that shape these associations. This comparative approach would enrich our understanding of the dynamics of variables and their implications for organizational outcomes.

Fifth, conducting intervention studies to reduce job insecurity and enhance organizational commitment could offer practical insights for organizations. Implementing interventions to improve perceived organizational support or promote job involvement could help mitigate the negative effects of job insecurity and foster a more committed workforce. Evaluating the effectiveness of such interventions and identifying best practices for enhancing employee wellbeing and organizational commitment would be valuable for practitioners and H.R. professionals.

In summary, while this research has contributed valuable insights into the relationships between job involvement, perceived organizational support, job insecurity, and organizational commitment, it is essential to acknowledge the limitations and consider future research directions. Addressing these limitations and pursuing these avenues of further investigation will contribute to a deeper understanding of the phenomena under study and enhance the practical implications for organizations striving to foster employee engagement, reduce job insecurity, and promote organizational commitment.

### 10.2 Limitation

There are a couple of limitations in the present research. Firstly, this study is related to the sample characteristics. Although a stratified random sampling technique was employed to draw the sample from the private sector in Malaysia, the findings may be specific to this context and effective size indicated that there may be limitation in practical applications. Generalizing the results to other countries should be approached with caution. Future research could consider expanding the sample to include participants from diverse industries or countries to enhance the external validity of the findings.

The second limitation is associated with the cross-sectional design employed in this study. While the data collected at a single point in time provided valuable insights into the relationships between job involvement, perceived organizational support, job insecurity, and organizational commitment, it restricted the ability to establish causal relationships or determine the directionality of the observed associations. Furthermore, this research explored job insecurity as a mediator; hence, due to the cross-sectional nature of the data, caution should be exercised when interpreting the mediating role of job insecurity. The inability to establish a true mediation relationship based on cross-sectional data has remained a limitation of this study. Future research employing longitudinal designs would allow for a more rigorous examination of the causal and mediating effects with a clearer understanding of the underlying mechanisms.

Despite efforts to address common method variance, the reliance on self-report measures introduces the possibility of response biases. Participants’ tendencies to provide socially desirable or biased responses may have influenced the results. Utilizing multi-source assessments or incorporating objective measures could help mitigate these biases and improve the validity of the findings. Lastly, it is important to recognize the potential limitations associated with the generalizability of the findings. While the study was conducted within the private sector in Malaysia, different cultural contexts, organizational settings, or industries may yield different results. Replication studies across diverse samples and contexts are needed to enhance the generalizability of the findings and validate the robustness of the relationships examined.

## Data availability statement

The original contributions presented in the study are included in the article/supplementary material, further inquiries can be directed to the corresponding authors.

## Ethics statement

The studies involving humans were approved by the Research Ethics Committee of the University Kebangsaan Malaysia (RECUKM). The studies were conducted in accordance with the local legislation and institutional requirements. The participants provided their written informed consent to participate in this study.

## Author contributions

CH: Conceptualization, Formal analysis, Investigation, Methodology, Project administration, Resources, Software, Validation, Writing – original draft, Writing – review & editing. N-AA: Conceptualization, Investigation, Methodology, Resources, Supervision, Writing – review & editing. WW: Conceptualization, Methodology, Resources, Supervision, Visualization, Writing – review & editing. NZ: Conceptualization, Formal analysis, Methodology, Resources, Supervision, Validation, Writing – review & editing̣.
